# A New Quantitative Tool for the Ultrasonographic Assessment of Tendons: A Reliability and Validity Study on the Patellar Tendon

**DOI:** 10.3390/diagnostics14111067

**Published:** 2024-05-21

**Authors:** Isabel Albarova-Corral, José Segovia-Burillo, Miguel Malo-Urriés, Izarbe Ríos-Asín, Jesús Asín, Jorge Castillo-Mateo, Zeus Gracia-Tabuenca, Mario Morales-Hernández

**Affiliations:** 1 PhysiUZerapy Health Sciences Research Group, Department of Physiatry and Nursing, University of Zaragoza, 50009 Zaragoza, Spain; 2 Fluid Mechanics, Instituto de Investigación en Ingeniería de Aragón (I3A), University of Zaragoza, 50018 Zaragoza, Spain; 3 Modelos Estocásticos Research Group, Department of Statistical Methods, University of Zaragoza, 50009 Zaragoza, Spain

**Keywords:** mathematical modeling, psychometric properties, quantification, tendinopathy, texture analysis, ultrasound

## Abstract

Ultrasound is widely used for tendon assessment due to its safety, affordability, and portability, but its subjective nature poses challenges. This study aimed to develop a new quantitative analysis tool based on artificial intelligence to identify statistical patterns of healthy and pathological tendons. Furthermore, we aimed to validate this new tool by comparing it to experts’ subjective assessments. A pilot database including healthy controls and patients with patellar tendinopathy was constructed, involving 14 participants with asymptomatic (*n* = 7) and symptomatic (*n* = 7) patellar tendons. Ultrasonographic images were assessed twice, utilizing both the new quantitative tool and the subjective scoring method applied by an expert across five regions of interest. The database contained 61 variables per image. The robustness of the clinical and quantitative assessments was tested via reliability analyses. Lastly, the prediction accuracy of the quantitative features was tested via cross-validated generalized linear mixed-effects logistic regressions. These analyses showed high reliability for quantitative variables related to “Bone” and “Quality”, with ICCs above 0.75. The ICCs for “Edges” and “Thickness” varied but mostly exceeded 0.75. The results of this study show that certain quantitative variables are capable of predicting an expert’s subjective assessment with generally high cross-validated AUC scores. A new quantitative tool for the ultrasonographic assessment of the tendon was designed. This system is shown to be a reliable and valid method for evaluating the patellar tendon structure.

## 1. Introduction

Tendons are anatomic structures that transmit the force generated by muscle to bone [1]. Tendinopathy is a major problem in the general population, being especially important in the working and athletic populations, accounting for up to 30–50% of their clinical consultations [2,3,4]. Furthermore, tendinopathies represent an important socioeconomic problem, involving annual costs above EUR 800 for each patient [5,6]. The most frequently affected tendons are the Achilles, rotator cuff, and patellar tendons [7,8,9]. These tendons, due to their anatomical and biomechanical characteristics, are usually subjected to high loads, with various vectors of action [10,11]. Moreover, these tendons usually present hypovascular or avascular areas, called watershed regions, with less regenerative capacity and therefore a greater predisposition to presenting pathology [12].

Patellar tendinopathy is defined as a clinical condition characterized by pain or discomfort in the region of the patellar tendon. It is related to activities such as jumping, running, or squatting, reducing its functionality. Patellar tendinopathy is also known as jumper’s knee due to its high prevalence in athletes who engage in sports that involve jumping, including volleyball or basketball [3]. In fact, jumper’s knee is the most prevalent pathology in volleyball and basketball players, with a prevalence of 40% [13,14], and tendon damage affects nearly 100% of elite players [15,16]. However, among young players (14–18 years of age), its prevalence has been shown to be lower [17,18], attributable to their relatively shorter exposure to cumulative loads [19,20]. Additionally, tendinopathy among workers is a significant concern due to its association with substantial economic costs stemming from productivity loss and compensation claims, with an incidence as high as 40% in certain professions [21]. This finding emphasizes the importance of early detection and prevention.

Ultrasound is considered the most suitable method for evaluating tendons since it allows the depiction of both main and secondary fiber bundles [22]. Ultrasonography has been shown to be a reliable and valid tool for discerning the anatomical details of superficial structures such as the patellar tendon [23]. It has been demonstrated that tendons with tendinopathy present changes in echogenicity as well as tendon size and thickness [18,19,24]. Patellar tendon thickness is a clinically relevant measure for assessing patellar tendinopathy [25]. In addition, it allows the evaluation of clinical evolution since a reduction in the thickness of the patellar tendon arises after successful treatments [26]. However, there is still debate about the typical sonographic appearance of this tendon, its alterations in pathological situations, and the presence of anomalies in asymptomatic subjects [13,16,18,27].

Ultrasonography is considered a safe, non-invasive, inexpensive, portable, non-irradiating method for the assessment of tendon structure in clinical practice [22]. According to certain authors, ultrasound evaluation is required for the diagnosis of tendinopathy [28]. However, this assessment tends to be subjective, with examiner dependency being the primary limitation for the implementation and interpretation of ultrasound [29]. To overcome this, different attempts have been made to quantify the ultrasonographic assessment of the tendon [30,31,32,33]. Van Schie et al. [30] compared symptomatic and asymptomatic Achilles tendons by an ultrasonographic tissue characterization (UTC) procedure and demonstrated that UTC can quantitatively evaluate tendon structure and thereby discriminate symptomatic and asymptomatic tendons. Bashford et al. [31] achieved in 2008 classify Achilles tendon images into tendinopathy and control categories by extracting eight spatial frequency parameters from regions of interest in tendon images, which were then filtered and classified using linear discriminant analysis. Other studies tried to quantify different parameters as tendon microvascular volume (MV) or cross-sectional area (CSA), demonstrating the ultrasound’s potential for both detecting microvascular changes in tendinopathy and reliably assessing tendon structural parameters [32,33]. However, many of these techniques are not widely available or are difficult to perform.

The main purpose of our study was to develop a new tool that allows us to quantify the ultrasonographic evaluation of the tendon. Furthermore, an example was developed and applied in the exploration of healthy and pathological patellar tendons in order to show this tool’s usefulness, reliability, and validity. Our hypothesis was that this new tool could offer quantitative data based on mathematical analysis that can be used to predict the subjective assessment of a trained professional when grading tendon injuries.

## 2. Materials and Methods

**Figure 1:**
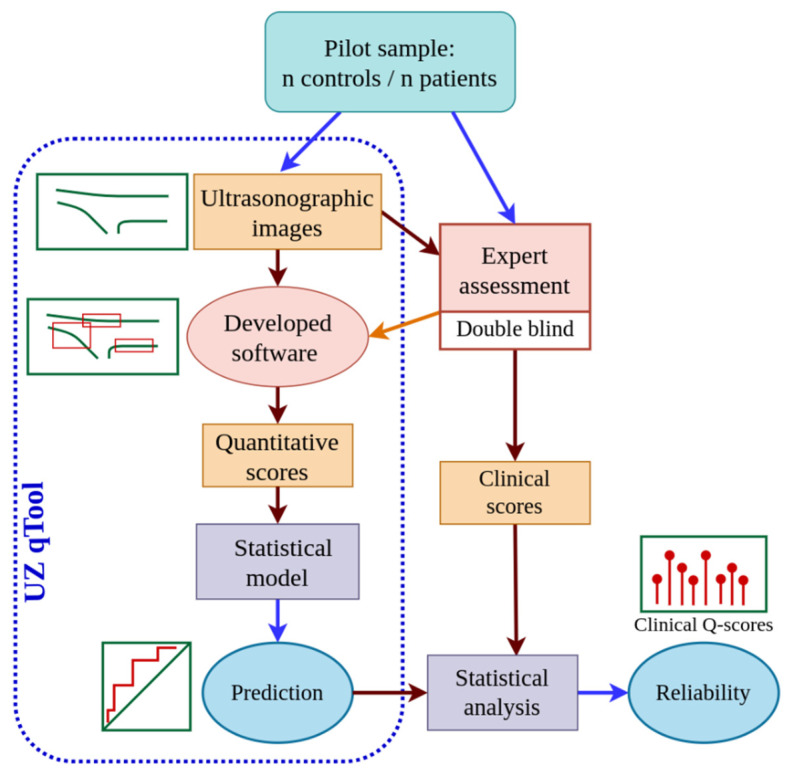
Workflow diagram of the study procedure.

This study was performed in four phases (Figure 1): (a) ultrasound scanning of the patellar tendons of the participants; (b) design of the quantitative assessment tool for ultrasound images of the patellar tendon, i.e., “UltraZound quantitative Tool (UZ qTool)”; (c) subjective and objective analysis of ultrasound images by an expert; and (d) statistical analysis of the software output. The fourth phase, described below in more detail, involved evaluating the reliability of the subjective and objective scores extracted from the ultrasound imaging and the prediction accuracy of the software output. Figure 1 shows a workflow diagram.

This study was designed according to the principles of the Declaration of Helsinki and approved by the Local Ethics Committee, and participants signed a written informed consent form prior to their inclusion in this study. Voluntary participants were recruited from patients attending a private clinic via an informative poster and social media. Fourteen patients (seven with patellar tendinopathy and seven healthy controls) participated in this study (Table 1). Participants were between 18 and 65 years of age. Healthy controls had no previous pathology or symptomatology in the knee.

### 2.1. Ultrasonographic Scanning of the Patellar Tendons of the Participants

Fourteen patellar tendons were evaluated with ultrasonography (*n* = 7 healthy; *n* = 7 pathological). Images were collected from the symptomatic tendons of individuals with patellar tendinopathy, all experiencing symptoms on their dominant side. For the healthy controls, images were also obtained from their dominant side. Ultrasonography of the patellar tendon was performed with the subject in the supine position, with a small support placed under the popliteal fossa to obtain a knee flexion angle of 30° [34]. Before the evaluation, the subjects were allowed a 5 min period of rest in a comfortable position to minimize tension on the patellar tendon [35].

First, the inferior pole of the patella was identified through palpation and was used as a reference point for ultrasound examination [34]. A probe was then placed slightly distal to the apex of the patella, longitudinal with respect to the patellar tendon, with the notch oriented proximally [35]. B-mode ultrasonography was recorded so that the results could be analyzed offline using the new designed system. The most affected part of the tendon was selected for evaluation. If no pathological region was detected, the proximal area of the tendon was measured 5 mm distal to the apex of the patella, as this region is commonly associated with pathological changes [36]. Ultrasound scanning was performed by the same professional, who had more than 10 years of experience in musculoskeletal ultrasound.

### 2.2. Design of the Quantitative Assessment Tool for Ultrasonographic Images of the Patellar Tendon

A program was designed and developed to quantitatively analyze ultrasound images of the tendon, a goal widely desired in the field of medical ultrasound [37,38]. The digital evaluation of ultrasonographic images necessitates a computational framework capable of parsing pixels within a medical image and computing representative values to assess tendon status. Echograms and various medical files are typically stored in the DICOM format, which can be easily converted to PNG for improved user-friendliness. This conversion may result in a loss of resolution but does not compromise the essential tendon-related image data.

Python (v3.10.12) served as the primary programming language for this tool due to its extensive range of operations and libraries. These include popular options like OpenCV (v4.9.0.80) [39] and PyFeats (v1.0.1) [40], as well as the ability to perform mathematical operations on matrices.

#### 2.2.1. Regions of Interest (ROIs)

**Figure 2:**
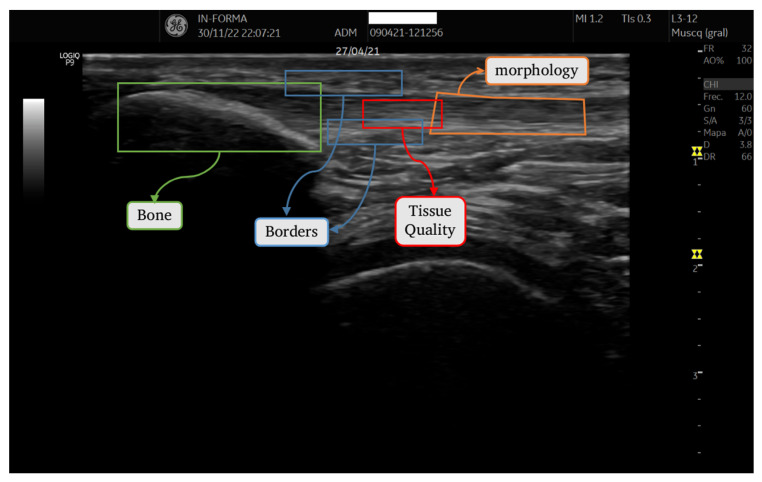
ROIs corresponding to each section.

To develop the quantitative tendon analysis tool, we considered the criteria assessed by experts when examining an ultrasound image of a tendon: bone insertion (enthesis), tendon borders (upper and lower peritendon), tendon tissue quality, and tendon thickness or morphology. Accordingly, our software was segmented into four distinct modules, as depicted in Figure 2. Each module operates on a separate region of interest (ROI) selected by the user. Within each ROI, a series of operations generates a specific descriptor.

#### 2.2.2. Quantifying Characteristics of ROI Images

Each module incorporates a suite of variables, encompassing both descriptive statistical indicators (such as maximum, minimum, mean, and standard deviation) and advanced image analysis techniques. These techniques include the Gray-level Co-occurrence Matrix (GLCM), Gray-level Difference Statistics (GLDS), and Haar Wavelet transforms [41]. For a comprehensive understanding, a brief elucidation of these advanced indicators is warranted.

- Gray-level Co-occurrence Matrix (GLCM): GLCM quantitatively characterizes the frequency of pixel intensity pairings at specified distances and orientations within an image. From this matrix, a variety of textural features can be extracted, including contrast, mean value, and variance. These features facilitate the analysis of the spatial patterns and textural properties of tissue.
- Gray-level Difference Statistics (GLDS): GLDS compute the statistical differences between pixel values along specified directions. These computations yield a matrix that provides insights into the texture of an image through metrics such as average intensity differences, contrast, homogeneity, and correlation. These metrics are instrumental in evaluating the spatial distribution of and variation in pixel intensities.
- Haar Wavelet Transform: This transform is employed to detect and analyze local variations in pixel intensity. By capturing gradients within an image, it effectively highlights areas of homogeneity and delineates regions with abrupt transitions in pixel values. This feature is particularly useful for identifying structural boundaries, such as those present at the edges of bone tissue.

The integration of these sophisticated image-processing techniques ensured a robust and multifaceted analysis of tendon and bone tissues, crucial for accurate diagnosis and evaluation. A detailed breakdown of each module is presented. Additionally, Table 2 summarizes the variables defined for each ROI.

Tissue Quality Analysis: This module utilizes pixel-based measurements such as mean intensity and standard deviation, predominantly focusing on texture analysis. Key techniques include GLCM and GLDS, whose efficacy is supported by prior research [4]. These analyses yield various metrics, such as contrast and variance, providing a comprehensive assessment of tissue quality.

Morphological Assessment of Tendons: This module focuses on a tendon’s geometry, including width, convexity, and parallelism. Unlike the rectangular ROI selection method used in the previous index, we request users to select a polygonal ROI that matches the tendon’s shape, enabling accurate measurements of distances and angles. Calculations of maxima, minima, means, and ratios, as well as estimations of parallelism and convexity, are derived from this geometric analysis. An estimation of parallelism is determined from the standard deviation, while convexity is calculated as the ratio between a ROI’s area and its convex hull area.

Bone Tissue Analysis: This module focuses on the bone into which the tendon is inserted. Recognizing a bone’s impact on tendon health, especially at the interface between the two, this module isolates the bone in question within a rectangular ROI using ultrasonographic reflectivity criteria. Key parameters include area, perimeter, convexity, and the number of bone fragments, which influence tendon health. The size (area and perimeter) and homogeneity of the bone are also crucial indicators of overall tissue health. For this objective, a thresholding process is performed to delineate the bone region [42].

Comprehensive details of all parameters are listed in Table 2.

### 2.3. Subjective and Objective Analysis of Ultrasonographic Images by the Expert

The expert subjectively and objectively evaluated the 14 patellar tendon (*n* = 7 healthy; *n* = 7 pathological) ultrasound images twice using the new tool. The assessments were conducted on separate days to minimize recall bias. The expert evaluator had more than 10 years of experience in tendon ultrasound and was blinded to the clinical statuses of the participants.

#### 2.3.1. Subjective Analysis of Ultrasonographic Tendon Images by the Expert

The subjective evaluation was performed using a four-grade scale, a modification of the score proposed by Sunding et al. [43]: 0—normal structure; 1—light structural changes; 2—moderate structural changes; and 3—severe structural changes (Table S1 of Supplementary Material). The expert applied this subjective scale to four parameters of the ultrasonographic images of the tendon: (a) bone insertion (enthesis), (b) tendon borders or interface (upper and lower epitenon), (c) tendon quality (tissue quality), and (d) morphology.

#### 2.3.2. Objective Analysis of Ultrasonographic Tendon Images by the Expert Using the New Tool

Independently, the expert used the newly developed tool for objective tendon analysis. They simply selected regions of interest in each tendon, enabling the new system to provide quantitative values for each image. The result of applying the software to an ultrasonographic image of the tendon was a vector with 51 variables.

### 2.4. Statistical Analysis of the Software Output

Each computation was conducted within the designated ROI for the tissue region, and the output, along with the evaluator’s subjective assessments, was documented for further statistical analysis.

For each ROI, we assessed the intra-rater reliability of the evaluator’s responses using a scale ranging from 0 to 3 and of the quantitative scores from the software output. Reliability was evaluated by means of the squared-weighted Kappa coefficient for the responses and the intraclass correlation coefficient (ICC) for the quantitative scores. Additionally, we calculated Kendall’s concordance coefficient (KCC or Kendall’s W) for all variables to directly compare the rank-based reliability between the ordinal responses and the quantitative variables. These coefficients range between 0 (no agreement) and 1 (total agreement). Kappa coefficient may assume negative values, but the latter interpretation remains between 0 and 1. Coefficients were fitted using the R package ‘irr’ (v. 0.84.1) [44].

#### Prediction Models

A prediction model is built for each ROI. The aim is to estimate probabilities of tendon status for a new patient from the image information. For each ROI, we defined a binary response variable based on whether the evaluator’s assessment was equal to three (Y3) or not. Y3 responses were fitted using logistic regressions via generalized linear mixed-effects models (GLMM) with fixed-effects linked to the image measurements and participants’ effect as random-effects. The GLMM effectively accounts for the intra- and inter-variability of the study design. Furthermore, it is difficult to detect non-linear patterns given a pilot sample size, then logistic regression models offer an interpretable robust approach preferable to other complex machine learning algorithms in this context. The GLMM model can be defined as the log odds ratio:

(1) $$ln\left(\frac{P({Y3}_{ij}=1\;|\;x_{ij},\;u_{j})}{P({Y3}_{ij}=0\;|\;x_{ij},\;u_{j})}\right)=\beta_{0}+\beta_{1}x_{ij}+u_{j}$$

where *Y*3*_ij_* stands for the dichotomized assessment response of the *i*’th observation of the *j*’th subject (i.e., level); *x_ij_* represents the quantitative covariate; *β*_0_ stands for the intercept; *β*_1_ is the regression coefficient; *u_j_* is the random effect of the j’th level. It is assumed that *u_j_* follows a normal distribution with mean zero and variance *σ*^2^. Then, the regression coefficient *β*_1_ measures the effect of increase/decrease in *x* by one unit on the log odds ratio [45].

First, quantitative variables of the ROI with an intraclass correlation coefficient (ICC) lower than 0.75 were rejected as explanatory variables for the models and discarded, considering them inadequate for establishing the classification procedure. Second, for each non-discarded variable, a GLMM with a single-term fixed effect was considered; this restriction is applied to avoid overparameterization in models due to the sample size. The estimation procedure uses the default parameter settings of the R package ‘lme4’ (v. 1.1-33) [46]. Then, we tested model prediction skills using a leave-one-level-out cross-validation (LOLO-CV) approach. The LOLO-CV works as follows: for each participant’s assessments, a GLMM is fitted with all the remaining participants as training data, and then we predict their responses using the GLMM fixed-effects model (i.e., GLMM prediction with a new random-effect level). Therefore, in each ROI binary response, for each quantitative variable, we calculated receiver operating characteristic (ROC) curves and computed their corresponding area-under-the-curve (AUC) scores under cross-validation conditions. The ROC expresses the trade-off between the sensitivity and specificity scores, and the AUC accounts for the area below of this curve. The AUC provides a comprehensive assessment of the selected model’s performance and reflects the model’s ability to predict different samples. It is one of the most frequent metrics for the assessment of model performance in practical applications [47,48]. The R package ‘pROC’ (v. 1.18.0) [49] was used. As a general reference for the AUC scores in the biomedical context, 0.5 can be interpreted as the nominal chance classification, while scores above 0.7 can be interpreted as fair prediction performance, those above 0.8 can be interpreted as good prediction performance, and those above 0.9 can be interpreted as excellent prediction performance [50,51]. Finally, the model with the highest AUC is selected.

Additionally, we developed the algorithm for a binary response defined based on whether the evaluator’s assessment was higher than zero (Y1) or not (i.e., an indicator of any type of structural changes).

## 3. Results

### 3.1. Experimental Procedure and Database

The database was obtained from the experimental/empirical use of an image method applied by an expert and designed to be applied to seven healthy controls and seven patients with patellar tendinopathy. Given that we had between 5 and 13 variables for each of the four ROIs and recorded multiple assessments for each ultrasonography, the cumulative result was a substantial volume of stored data.

The expert’s 0-to-3 classification for the images is summarized in Table S2 of the Supplementary Materials. As shown, light structural changes were found in some ROIs in healthy participants, while some ROIs in participants with tendinopathy were scored as 0, without apparent pathological alterations. Approximately half of the scores for Bone, Thickness, and Upper Edge ROIs were graded as 0, showing a more conventional pattern compared to the scores obtained for Quality and Lower Edge ROIs, where the rating was more widespread. In fact, no ratings of 0 were obtained for the lower-edge ROI.

Table S3 in the Supplementary Materials summarizes the description of the image measurements in every ROI for the groups defined by the expert classification for 0 and 3 levels of tendinopathy. Overall, the average values of the quantitative variables contrasted between these two levels; in particular, Bone and Quality ROI values tended to differ more than their corresponding standard deviations.

### 3.2. Reliability Scores

**Figure 3:**
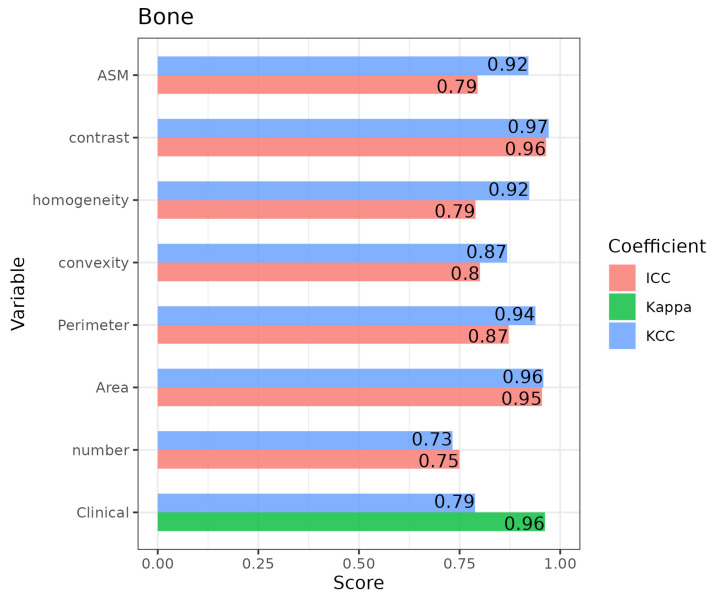
Bar plot including the intra-rater coefficient scores for the Kendall’s concordance (KCC), Kappa, and intraclass correlation (ICC) coefficients for the intra-rater reliability for the “bone” ROI.

The reliability of the clinical assessments through the Kappa and KCC scores was analyzed. Table 3 and Figure 3 show the results obtained for one of the ROIs, namely, the Bone ROI, with the label ‘Clinical’. Figures S1–S4 in the Supplementary Materials summarize the results for the other ROIs. In general, a high level of agreement was obtained for most of the ROIs, with Kappa scores over 0.9 and KCC scores above 0.75. A different trend was identified for the Lower-Edge ROI, with moderate scores (Kappa = 0.67; KCC = 0.51).

Regarding the reliability of the quantitative variables assessed using ICCs, all variables for the Bone ROI showed high levels of agreement, with some scores being over 0.9 and all of them being over 0.75 (Figure 3). The results for the rest of the ROIs appear in Figures S1–S4 in the Supplementary Materials. Nevertheless, all scores were over 0.75 for Quality ROI and for most of the Lower and Upper Edges variables. Regarding the Thickness ROI, only two measurements had ICC scores over 0.75. In contrast, the KCC scores for the quantitative variables tended to be higher than the ICCs, which implies that variable units differed to a greater extent than the variable ranks.

### 3.3. Prediction Scores

**Figure 4:**
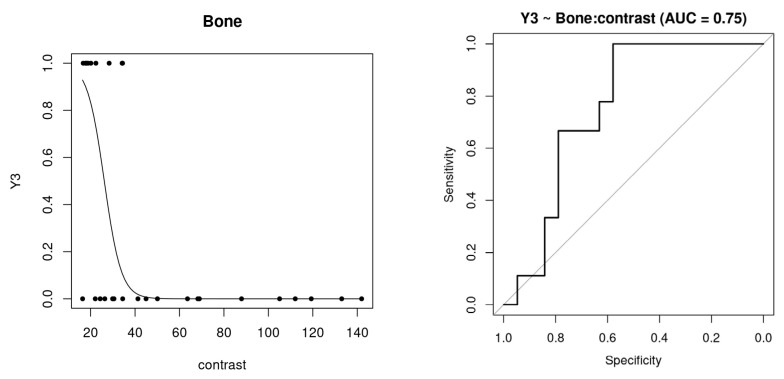
Logistic regression curve for the GLMM model of the binary response Y3 vs. quantitative variable “Contrast” of the Bone ROI (**left**) and receiver operating characteristic (ROC) curve (**right**) for its prediction responses of the Leave-one-level-out Cross-validation (LOLO-CV).

The prediction results based on the quantitative variables showed, in general, relatively high AUC scores for the prediction of the evaluation responses. Table S4 of the Supplementary Materials summarizes the results for the different ROIs. Specifically, for the Bone ROI, the prediction for assessment indicating some change (Y1, light to severe change) shows high AUC scores of over 0.9 for homogeneity and ASM measures. Lower scores were obtained for the prediction of severe change (assessment of the evaluation of level three, Y3), but contrast measurement has an associated AUC of 0.75 (see Table 3). In particular, Figure 4 shows the prediction function based on contrast measurement (left panel), as well as the corresponding ROC curve (right). Both the Thickness and Quality ROIs showed excellent AUC scores, with values over 0.89 for both responses Y1 and Y3 (see Supplementary Materials, Table S4). Therefore, in these ROIs, whichever image variable had a high predictive ability. For instance, Quality’s GLDS_homogeneity was able to classify in both health and severe conditions with high accuracy. The Upper Edge ROI showed excellent ability for the prediction of healthy controls and for some of the GLCM variables, particularly GLCM_IDmoment. However, it was not possible to predict the Y3 response, because none of the patients scored level 3 in both repetitions, so the corresponding model was not created.

## 4. Discussion

Ultrasonography is widely used in clinical practice for the assessment of tendons as it offers advantages such as safety, cost-effectiveness, and ease of application. However, operator dependency and subjectivity of interpretation are the major limitations to its widespread utility. Our study aimed to develop a new tool for quantifying ultrasonographic assessments of the patellar tendon. Moreover, we aimed to test its reliability and validity in order to explore its usefulness to evaluate pathological changes in tendon structure and its applicability to clinical practice. Particularly, we plan to broaden the tool’s applicability to other tendons like the Achilles tendon or the plantar fascia tendon to gain valuable insights into its utility across different anatomical regions.

A new quantification assessment tool for sonographic images of tendons was developed. It provides several variables concerning four different ROIs in tendinopathies. Other authors have developed systems for the ultrasound quantification of the tendon, with particular emphasis on Ultrasound Tissue Characterization (UTC). These systems have shown fair-to-good intra-examiner reliability (ICC = 0.61–0.88), being comparable to the values obtained in our study. The tool developed in the current study not only provides a quantitative assessment of the tissue quality of a tendon but also evaluates bone insertion, tendon borders, and morphology, which are ROIs that should be explored in sonographic assessments of tendinopathies according to Sunding et al. [43].

A high level of reliability was found for most of the ROIs, with Kappa scores over 0.9, KCC scores above 0.75, and ICCs over 0.75 for Quality ROI and most of the Lower and Upper Edges variables. Previous studies on sonographic assessment found higher reliability of quantitative measurements compared to qualitative measurements [52]. According to the literature, thickness and cross-sectional area (CSA) are the most commonly used quantitative measures in tendon ultrasound assessment. Several studies evaluated the reliability of these measurements for different tendons [18,29,53,54], with positive results for intra- and inter-rater reliability, especially with respect to expert raters [26,52]. Skou et al. [26] found high intra- (ICC = 0.89–0.94) and inter-rater (ICC = 0.78–0.91) reliability of tendon thickness and CSA measurements in the patellar tendon [26,33].

A high ability for prediction was obtained in most of the ROIs: both Thickness and Quality ROIs showed excellent AUC scores over 0.89 for both responses Y1 and Y3; the Upper Edge ROI manifested excellent ability for the prediction of healthy controls; and Bone ROI obtained AUC scores over 0.9 for assessment Y1. The ability to predict changes in tendons with quantitative measures has previously been explored in regard to the patellar tendon. Ríos-Díaz et al. [25] found that the quantification of thickness and CSA turned out to be a good indicator of patellar tendinopathy. Thickness and CSA are simple and practical measurements in clinical practice, but they may be insufficient for detecting subtle or subclinical tissue changes [17,20]. To the best of our knowledge, this is the first approach in the literature that aims to provide quantitative reliable information on the four ROIs to assess tendon condition with ultrasonography.

Finally, the following limitations must be recognized. Firstly, the assessment was performed solely on the patellar tendon. This is a superficial, relatively wide, linear, and thick tissue, making it easy to assess with ultrasonography. Moreover, patellar tendinopathy is one of the most prevalent pathologies in tendons in the general population, making it clinically relevant. It was purposefully chosen to facilitate the initial validation of this new tool. Therefore, some precautions must be considered when extrapolating the results to other tissues. The initial sample size included seven tendons with patellar tendinopathy and seven healthy controls. In a further step, our study will expand by increasing the subject sample size to refine the evaluation tool. By including a more diverse cohort, we aim to enhance the reliability and validity of our quantitative assessment of tendon pathologies. Secondly, ultrasonographic assessment is a method that depends on the interpretation of the examiner, hindering the quantification of objective variables. Previous studies have shown higher reliability and validity values for experienced examiners than non-experienced examiners in regard to ultrasound assessment [33,55]. Thus, the evaluator chosen for our study was a professional with more than ten years of experience in musculoskeletal ultrasonography. Furthermore, a standardized protocol was followed to obtain precise and reliable measurements and avoid the main difficulties that crop up when performing ultrasound. This includes minimizing probe pressure, which can influence size measurements, and tilting the probe perpendicular to the tissue to avoid image distortion [56,57]. To overcome this limitation, the authors recommended that a single evaluator with experience should be employed to capture all images [58]. Thirdly, although a new easy ultrasound quantification tool has been developed and is available for any ultrasound machine, in the present study, only one ultrasound machine was used. Given the large number of ultrasound machines available, the quantified measurements should be comparable from one machine to another to allow higher practicality [33,59]. In this regard, previous authors demonstrated that tendon measurements are reliable, even when obtained with different pieces of equipment [33,59]. Fourthly, certain limitations concerning the statistical modeling should be considered. Particularly, the relatively low sample size may have impacted the accuracy of the intra-rater reliability and the performed prediction analyses. These limitations should be considered when interpreting the results of our study. Therefore, in future studies, we aim to address these limitations by increasing both the sample size and the diversity of the cohort to enhance the accuracy of analytical predictions. Additionally, as we refine the tool for patellar tendon assessment, we will expand our investigations to include other tissues, such as the Achilles tendon and the plantar fascia

## 5. Conclusions

A new tool for quantifying the ultrasonographic evaluation of tendons was developed and validated for the patellar tendon. This new tool showed high reliability, with ICCs above 0.75 in most cases. Several quantitative variables manifested an ability to predict the expert’s subjective assessment. These results evidenced, in general, relatively high AUC scores for the prediction of the evaluation responses. The results should be interpreted in consideration of the limitations of this study.

## Figures and Tables

**Table 1:** Demographic characteristics of the sample. Continuous variables include means (±standard deviation).

	Healthy Controls	Patients
Size	7	7
Sex (F/M)	3/4	3/4
Age (y.o.)	39.6 (±13.2)	37.4 (±14.2)
Height (cm)	172.1 (±7.5)	172.4 (±9.3)
Weight (kg)	71.4 (±8.9)	69.1 (±11)

**Table 2:** List of the studied variables for each ROI.

Tendon Tissue Quality	Tendon Borders(Upper/Lower)	Tendon Morphology	Bone Tissue
GLCM Matrix Contrast GLCM Sum Average GLCM Sum-of-Squares Variance GLCM Difference Variance GLCM Correlation GLCM Inverse Difference Moment GLDS Homogeneity GLDS Contrast GLDS Angular Second Moment GLDS Entropy GLDS Mean Haar Mean Haar Variance	GLCM Matrix Contrast GLCM Sum Average GLCM Sum-of-Squares Variance GLCM Difference Variance GLCM Correlation GLCM Inverse Difference Moment GLDS Homogeneity GLDS Contrast GLDS Angular Second Moment GLDS Entropy GLDS Mean Haar Mean Haar Variance	Tendon Max Width Tendon Min Width Mean Width Standard Deviation (Parallelism) Ratio between Max and Min Width	Number of Bone Fragments Bone Area Bone Perimeter Convexity GLCM Homogeneity GLCM Contrast GLCM Correlation

**Table 3:** Area under the curve (AUC) of the receiver operating characteristic (ROC) curve for the prediction responses of the Leave-one-level-out Cross-validation (LOLO-CV), and estimated *β*1 parameter with its standard error (s.e.) for each quantitative variable of the Bone ROI and binary response Y3 (Evaluation = 3).

ROI: Bone	AUC	*β*_1_ (s.e.)
Area	0.63	22.79 (13.07)
Perimeter	0.57	2.09 (1.24)
Convexity	0.7	15.61 (49.02)
Homogeneity	0.63	7.42 (36.91)
Contrast	0.75	−0.26 (0.22)
ASM	0.67	3.02 (19.14)

## Data Availability

The data presented in this study are available on request from the corresponding author.

## Supplementary Materials

The following supporting information can be downloaded at https://www.mdpi.com/article/10.3390/diagnostics14111067/s1. Table S1: Description of the subjective tendon assessment scale scores; Table S2: Subjective classification for each ROI by an expert in categories from 0 to 3; Table S3: Description of variables for each ROI in groups defined by expert classification in categories 0 or 3; Table S4: Area under the curve (AUC) of the receiver operating characteristic (ROC) curve for the prediction responses of the Leave-one-level-out Cross-validation (LOLO-CV) for each quantitative variable of the bone, thickness, and quality regions of interest and binary responses of Y1 (Evaluation ≥ 1) and Y3 (Evaluation = 3); Figure S1: Bar plot including the intra-rater coefficient scores for the Kendall’s concordance (KCC), Kappa, and intraclass correlation (ICC) coefficients for the intra-rater reliability in the “Thickness” region of interest; Figure S2: Bar plot including the intra-rater coefficient scores for the Kendall’s concordance (KCC), Kappa, and intraclass correlation (ICC) coefficients for the intra-rater reliability in the “Quality” region of interest; Figure S3: Bar plot including the intra-rater coefficient scores for the Kendall’s concordance (KCC), Kappa, and intraclass correlation (ICC) coefficients for the intra-rater reliability in the “Lower edge” region of interest; Figure S4: Bar plot including the intra-rater coefficient scores for the Kendall’s concordance (KCC), Kappa, and intraclass correlation (ICC) coefficients for the intra-rater reliability in the “Upper edge” region of interest.
