# Supplementary material for: A New Quantitative Tool for the Ultrasonographic Assessment of Tendons: A Reliability and Validity Study on the Patellar Tendon
Source: Diagnostics (Basel). 2024 May 21;14(11):1067. doi: 10.3390/diagnostics14111067 (PMC11171978; doi:10.3390/diagnostics14111067)
Supplement: Supplementary file 1 [file diagnostics-14-01067-s001.zip › diagnostics-2960408-supplementary.pdf]

Supplementary material

Table S1. Description of the subjective tendon assessment scale scores.

	0	1	2	3
**Bone insertion**	Well-defined linear cortical.	Well-defined continuous cortical but with small irregularities.	Moderate irregularities which imply some discontinuity.	Marked irregularities or exostosis.
**Tendon interface**	Clearly defined edges.	Well-defined edges, but with some blurred regions.	Large areas with poorly defined borders.	Severe difficulties in differentiating the edges or impossible to differentiate.
**Tendon quality**	Homogeneous echogenicity. Normal, parallel, well-marked fibrillar pattern without losses inside.	Discrete alterations in echogenicity. Areas of loss of normal hypoechoic pattern, without anechoic areas.	Clear hypoechoic or anechoic areas, but less than 50% of the surface.	Large hypoechoic or anechoic areas greater than 50% of the surface or calcifications.
**Morphology**	Normal and homogeneous shape of the tendon.	Slight increase in size less than 50%.	Moderate increase in size, but less than 100%.	Large increase in size, greater than 100%.

Table S2. Subjective classification for each ROI by an expert in categories from 0 to 3.

	**EXPERT CLASSIFICATION**			
**ROI**	**0**	**1**	**2**	**3**
Bone	15	1	3	9
Thickness	13	1	14	0
Quality	8	6	3	11
Lower edge	3	19	3	3
Upper edge	15	11	1	1

Table S3. Description of variables for each ROI in groups defined by expert classification in categories 0 or 3.

		Expert classif=0		Expert classif=3	
		**Mean**	**SD**	**Mean**	**SD**
Bone	Area	0.36	0.14	0.65	0.17
	Perimeter	4.64	1.48	7.27	2.2
	Convexity	0.83	0.1	0.78	0.15
	Homogeneity	0.79	0.06	0.82	0.04
	Contrast	69.65	42.44	23.41	7.07
	ASM	0.51	0.11	0.57	0.07
Thickness	Maximum	0.44	0.06	0.88	0.19
	Minimum	0.38	0.08	0.77	0.14
	Mean	0.41	0.07	0.84	0.17
	X1.std	0.04	0.05	0.04	0.03
	Ratio	0.88	0.11	0.88	0.08
Quality	GLCM_Contrast	34.21	9.85	24.97	4.46
	GLCM_SumAverage	178.63	44.34	95.73	3.86
	GLCM_SoSVariance	395.23	124.39	591.13	215.61
	GLCM_DVariance	0	0	0	0
	GLCM_Correlation	0.95	0.01	0.98	0.01
	GLCM_IDMoment	0.34	0.02	0.36	0.02
	GLDS_Homogeneity	6053.62	1366.2	28284.38	7630.98
	GLDS_Contrast	584133.8	199427.8	1882140	588670
	GLDS_ASM	51961703	22411100	1075366664	530408474
	GLDS_Entopy	-132460.7	32825.2	-702269	210969
	GLDS_Mean	64356.03	15560.98	241608.6	70057.84
	Haar_mean	179.06	44.06	95.51	3.85
	Haar_variance	1549.24	488.27	2357.83	872.54
Lower edge	GLCM_Contrast	28.35	10.15	21.67	4.61
	GLCM_SumAverage	139.12	38.2	100.03	19.27
	GLCM_SoSVariance	484.64	131.75	465.6	150.95
	GLCM_DVariance	0	0	0	0
	GLCM_Correlation	0.97	0.02	0.97	0.02
	GLCM_IDMoment	0.33	0.03	0.37	0.02
	GLDS_Homogeneity	6544.25	3989.57	14297.63	5288.19
	GLDS_Contrast	487486.1	189187.7	779761.3	189970.2
	GLDS_ASM	67860910	73243098	273521173	159562213
	GLDS_Entopy	-144559.7	89073.58	-322465.3	121083.5
	GLDS_Mean	62052.75	25856.51	109744.6	32180.54
	Haar_mean	138.93	37.66	100.08	18.98
	Haar_variance	1928.49	520.6	1850.13	629.09

Table S3. (Cont.) Description of variables for each ROI in groups defined by expert classification in categories 0 or 3

		Expert classif=0		Expert classif=3	
		**Mean**	**SD**	**Mean**	**SD**
Upper edge	GLCM_Contrast	36.68	9.74	32.61	NA
	GLCM_SumAverage	163.02	58.29	114.34	NA
	GLCM_SoSVariance	491.94	169.5	297.53	NA
	GLCM_DVariance	0	0	0	NA
	GLCM_Correlation	0.96	0.01	0.94	NA
	GLCM_IDMoment	0.32	0.02	0.35	NA
	GLDS_Homogeneity	6025.09	1020.18	16850.57	NA
	GLDS_Contrast	664893.8	246212.4	1540994	NA
	GLDS_ASM	51606087	16293655	377513500	NA
	GLDS_Entopy	-136335.9	27633.7	-408488.2	NA
	GLDS_Mean	71115	18313.38	165990.8	NA
	Haar_mean	162.8	58.16	114.62	NA
	Haar_variance	1902.09	664.97	1162.96	NA

Figure S1. Barplot including the intra-rater coefficient scores for the Kendall's concordance (KCC), Kappa, and intraclass correlation (ICC) coefficients for the intra-rater reliability in the "Thickness" region of interest.

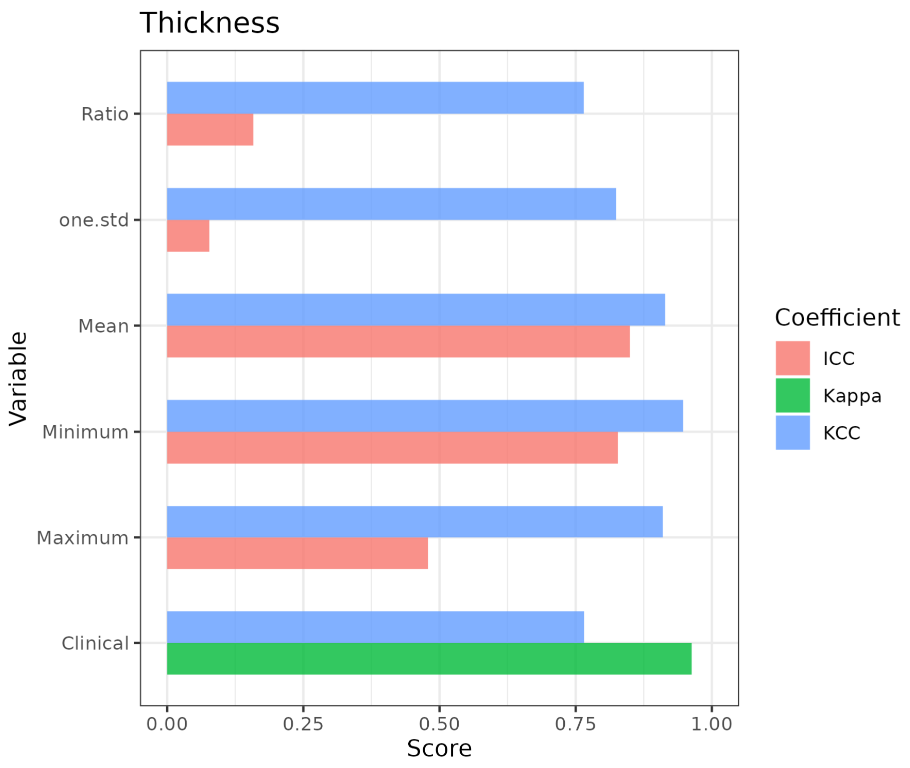

Figure S2. Barplot including the intra-rater coefficient scores for the Kendall's concordance (KCC), Kappa, and intraclass correlation (ICC) coefficients for the intra-rater reliability in the "Quality" region of interest.

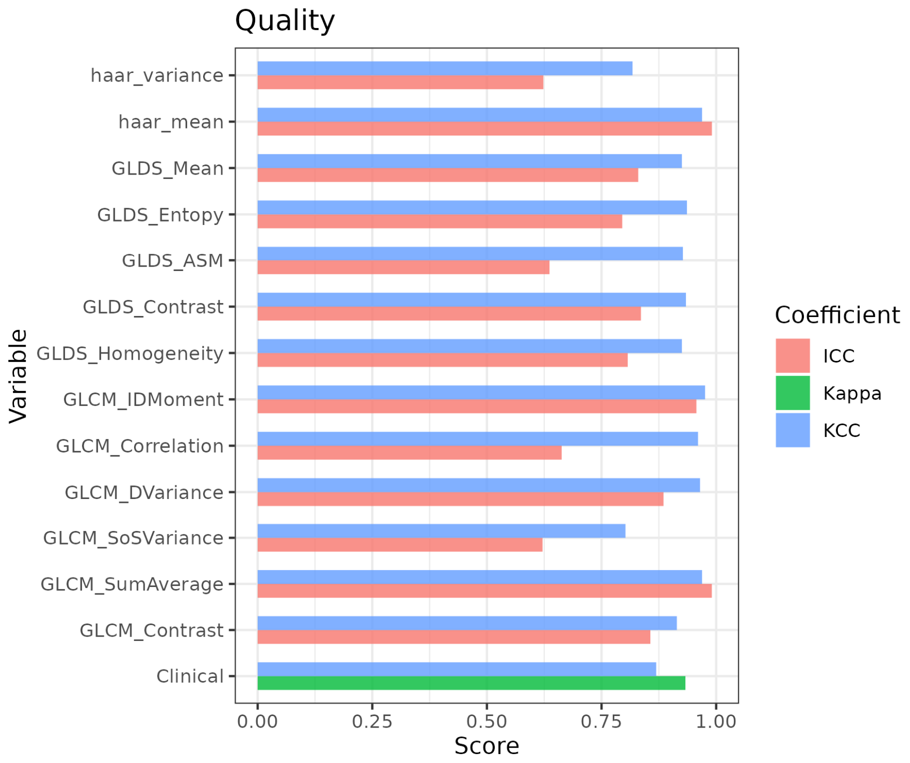

Figure S3. Barplot including the intra-rater coefficient scores for the Kendall's concordance (KCC), Kappa, and intraclass correlation (ICC) coefficients for the intra-rater reliability in the "Lower edge" region of interest.

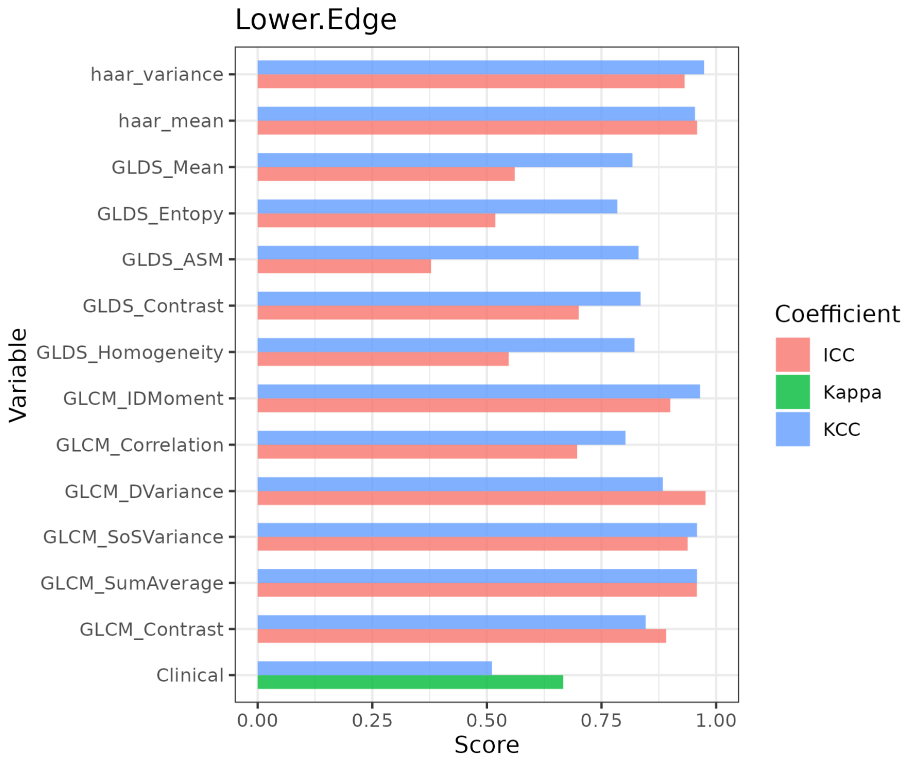

Figure S4. Barplot including the intra-rater coefficient scores for the Kendall's concordance (KCC), Kappa, and intraclass correlation (ICC) coefficients for the intra-rater reliability in the "Upper edge" region of interest.

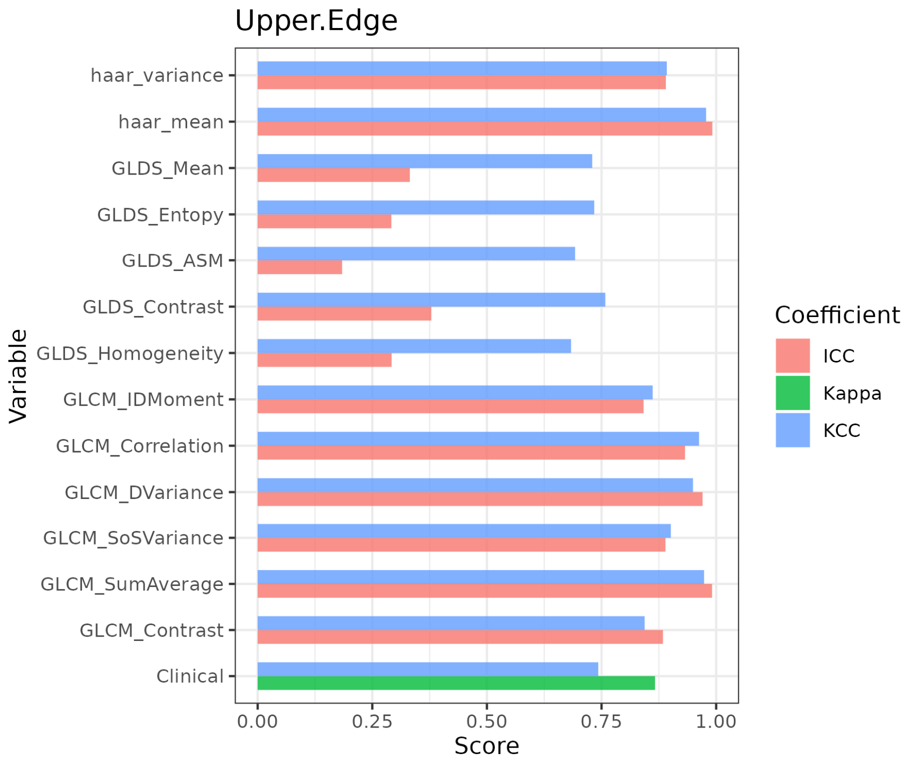

Table S4: Area-under-the-curve (AUC) of the receiver operating characteristic (ROC) curve for the prediction responses of the Leave-one-level-out Cross-validation (LOLO-CV) for each quantitative variable of the bone, thickness, and quality regions of interest and binary responses of Y1 (Evaluation >=1) and Y3 (Evaluacion = 3).

		Y1	Y3
Bone	Area	0.7461538	0.6315789
	Perimeter	0.7897436	0.5730994
	Convexity	0.6974359	0.6959064
	Homogeneity	0.9333333	0.6257310
	Contrast	0.7128205	0.7543860
	ASM	0.9333333	0.6783626
Thickness	Minimum	0.9358974	1
	Mean	1	0.9515306
Quality	GLCM_Contrast	0.4375	0.8128342
	GLCM_SumAverage	0.71875	NA
	GLCM_DVariance	0.8875	0.828877
	GLCM_IDMoment	0.5875	0.6684492
	GLDS_Homogeneity	0.88125	0.8823529
	GLDS_Contrast	NA	NA
	GLDS_Entopy	0.89375	0.8636364
	GLDS_Mean	0.9	0.842246
	Haar_mean	0.725	NA

Table S4 (Cont.). Area-under-the-curve (AUC) of the receiver operating characteristic (ROC) curve for the prediction responses of the Leave-one-level-out Cross-validation (LOLO-CV) for each quantitative variable of the upper and lower edge regions of interest and binary responses of Y1 (Evaluation >=1) and Y3 (Evaluation = 3).

		Y1	Y3
Upper Edge	GLCM_Contrast	0.7589744	NA
	GLCM_SumAverage	0.5589744	NA
	GLCM_SoSVariance	0.6153846	NA
	GLCM_DVariance	0.6666667	NA
	GLCM_Correlation	0.5025641	NA
	GLCM_IDMoment	0.9538462	NA
	Haar_mean	0.5589744	NA
	Haar_variance	0.6102564	NA
Lower Edge	GLCM_Contrast	NA	0.46875
	GLCM_SumAverage	NA	0.6302083
	GLCM_SoSVariance	NA	0.53125
	GLCM_DVariance	NA	0.4791667
	GLCM_IDMoment	NA	0.453125
	Haar_mean	NA	0.6302083
	Haar_variance	NA	0.5208333
